# Dynamic Interactions Between the Immune System and the Neuroendocrine System in Health and Disease

**DOI:** 10.3389/fendo.2021.655982

**Published:** 2021-03-22

**Authors:** John R. Klein

**Affiliations:** Department of Diagnostic Sciences, School of Dentistry, The University of Texas Health Science Center at Houston, Houston, TX, United States

**Keywords:** systemic regulation and adaptation, Hashimoto disease, hematopoiesis, thyroid hormones, integrated

## Abstract

The immune system and the neuroendocrine system share many common features. Both consist of diverse components consisting of receptors and networks that are widely distributed throughout the body, and both sense and react to external stimuli which, on the one hand control mechanisms of immunity, and on the other hand control and regulate growth, development, and metabolism. It is thus not surprising, therefore, that the immune system and the neuroendocrine system communicate extensively. This article will focus on bi-directional immune-endocrine interactions with particular emphasis on the hormones of the hypothalamus-pituitary-thyroid (HPT) axis. New findings will be discussed demonstrating the direct process through which the immune system-derived thyroid stimulating hormone (TSH) controls thyroid hormone synthesis and bone metamorphosis, particularly in the context of a novel splice variant of TSHβ made by peripheral blood leukocytes (PBL). Also presented are the ways whereby the TSHβ splice variant may be a contributing factor in the development and/or perpetuation of autoimmune thyroid disease (AIT), and how systemic infection may elicit immune-endocrine responses. The relationship between non-HPT hormones, in particular adipose hormones, and immunity is discussed.

## Introduction

In its most elemental form, homeostasis can be viewed as an integrated state of equilibrium between various physical and chemical processes, not only within individual organ systems, but also throughout the body overall. Whereas most biomedical research is conducted from a highly-focused reductionist perspective given the inherent difficulties in attempting to measure and quantify multifaceted processes, there is nonetheless a need to continually reflect on the vast cross-talk of organ systems in the body.

The immune system and the neuroendocrine system both consist of widely-distributed tissues, cells, receptors, ligands, and molecules. Moreover, both systems are highly adapted to sense external signals from the environment, and to communicate information regarding those throughout the body. It is perhaps not surprising, therefore, that the immune system and the neuroendocrine system interact broadly at many levels. In fact, the immune system and the neuroendocrine system collectively have been referred to as a “sixth sense” based on shared neuropeptides and neurotransmitters used by the immune system (1). One of many examples of this is the dynamic set of interactions between the immune system and the neuroendocrine system in the gut. In fact, there are at least three mechanisms for detecting changes in the intestinal wall, consisting of neural sensation delivered by extrinsic and intrinsic afferent neurons, more than twenty endocrine hormones produced by the cells of the mucosal epithelium, and immune responses to local and systemic antigens (2). Collectively, these form a web of communication and defense at the level of the gut. However, many other examples of this exist, as will be discussed in the following sections.

That TSH is produced by cells of the immune system was first reported almost forty years ago (3, 4). TSH is also produced by mouse intestinal crypt enterocytes and intestinal leukocytes, particularly in “hotblocks” of experimental rotavirus and reovirus infection (5, 6). Two sets of findings opened the way for understanding a potential role for immune system TSH. First, hypophysectomized mice that are unable to make pituitary TSH had elevated levels of T4 following alloantigen priming similar to that of non-hypophysectomized animals (7). Second, bone marrow (BM) hematopoietic cells and PBL were found to produce a novel splice variant of TSHβ (8), as discussed in detail below.

## Bidirectional Immune-Endocrine Interactions of the HPT Axis

The HPT axis is a critically-important hormone network for maintaining basal metabolism, growth, development, mood, and cognition. TSH is released into the circulation from the anterior pituitary followings thyrotropin releasing hormone (TRH) stimulation from the hypothalamus. TSH binds to and induces the release of the thyroid hormones (TH) thyroxine (T4) and triiodothyronine (T3) from the thyroid after binding to TSH receptors (TSHR), a seven-transmembrane domain G-protein coupled molecule on thyroid follicular cells. The majority of T4 is converted into the more biologically active T3 form following deiodination in target tissues after binding to thyroid hormone transporters (9, 10). The TSHR is also widely-distributed across many tissues outside the HPT axis (11).

Thyroid hormones have been shown to exert pleiotropic effects on PBL and on the inflammatory response. Early studies demonstrated that thymic peptides such as thymopoietin, thymulin, and thymosin produced by the thymic epithelium can have a positive effects on the secretion of hormones from the adenohypophysis (12). It was demonstrated in a series of studies that the thyroid is extensively involved in the maturation of the thymus (13–15). Conversely, THs have been shown to upregulate thymulin secretion (14). Exposure of T cells to TH has time dependent effects in that short-term exposure results in suppressed proliferation and apoptosis, whereas long-term exposure induces T cell proliferation. This appears to be regulated at least in part by activation of inducible nitric oxide synthetase (iNOS) (16–19). B cells respond differently to THs in that exposure induces development and cell-proliferation *in vivo* (20). T3 has direct effects on the maturation of macrophages into the M1 and M2 forms (21). T4 also has beneficial effects on the recovery from *Neisseria meningitidis* infection, mediated by iNOS production and nitric oxide mobilization (22). T4 blocks macrophage inhibitory factor proinflammatory activity *in vivo* and enhances survival of mice with induced sepsis (23, 24). The TSHR is expressed at high levels on a subset of murine dendritic cells (DCs), though it is minimally expressed on T cells and B cells. However, for reasons that are unclear, the TSHR is expressed on more lymph node T cells and B cells than on spleen cells (25). TSH enhances the phagocytic activity of DCs (25). TH have complex effects on the development and function of DCs, macrophages, and monocytes. Studies in which hypothyroid patients were treated with exogenous TH had increases in both plasmacytoid and myeloid DCs (26).

Adipose hormones such as adiponectin and leptin, which regulate metabolism and energy efficiency, also influence immunological function *via* receptors expressed on immune cells, particularly on M2-differentiated macrophages (27). Adiponectin has direct immunoregulatory activity by inhibiting the secretion of proinflammatory cytokines and increasing immunosuppressive cytokines (28, 29). Mice deficient in adiponectin fail to effectively modulate metabolic homeostasis (30). Leptin increases immune cell development, chemotaxis, and cytokine secretion (31, 32). Moreover, M1 and M2 macrophages in adipose tissues have opposing effects on insulin responses in that M1 macrophages promote insulin resistance whereas M2-macrophages enhance insulin sensitivity (33, 34). Invariant NKT (iNKT) cells and mast cells are present in adipose tissues (35, 36). Both of those are distinguished by their ability to rapidly respond to danger signals and to produce proinflammatory and regulatory cytokines. iNKT cells, in particular, are known to be a significant source of IFN-γ, IL-2, IL-4, IL-13, IL-17, and IL-21, as well as TNFα and GM-CSF, among others (37), all of which have important immunoregulatory activities and functions.

## A Novel TSHβ Isoform Produced by the Central and Peripheral Immune System

TSH is one of three glycoprotein hormones made in the anterior pituitary. All glycoprotein hormones share a common α-chain molecule and a unique hormone-specific β-chain component. TSHβ is highly conserved across many mammalian species. Until recently, no functional isoforms of TSHβ had been identified. We characterized a unique in-frame splice variant of TSHβ (referred to as TSHβv), which is copiously made by PBL and BM hematopoietic cells, in particular though not exclusively on myeloid cells (8, 38–40). Notably, TSHβv is stored in intracellular secretory vesicles in macrophages (39), a property that would facilitate rapid release under appropriate conditions. In that context, it will be interesting to define the signals that drive the release of intracellular TSHβv.

**Figure 1:**
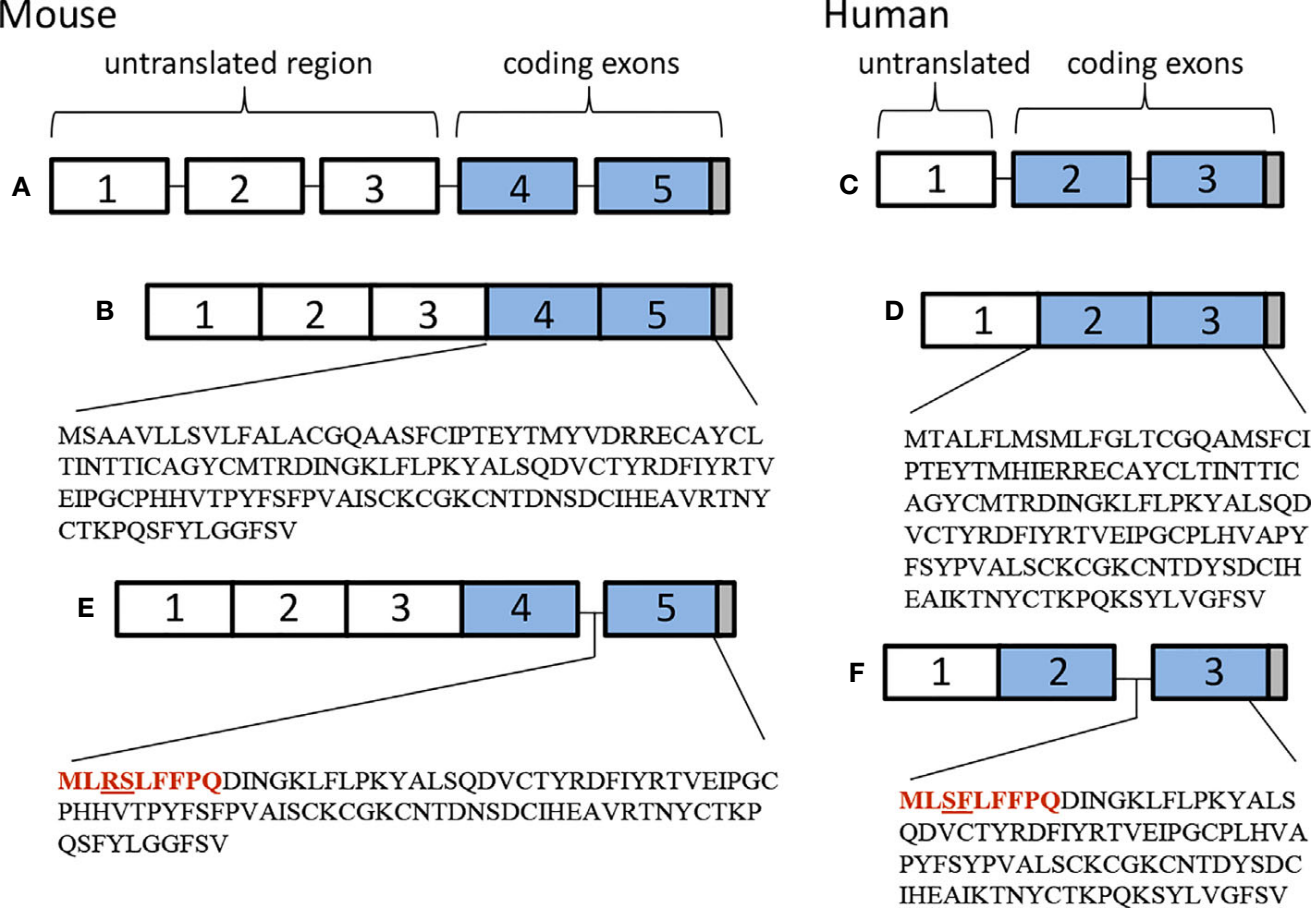
Genetic organization of **(A, B)** mouse and **(C, D)** human native TSHβ, and **(E)** mouse and **(F)** human TSHβv.

**Figure 2:**
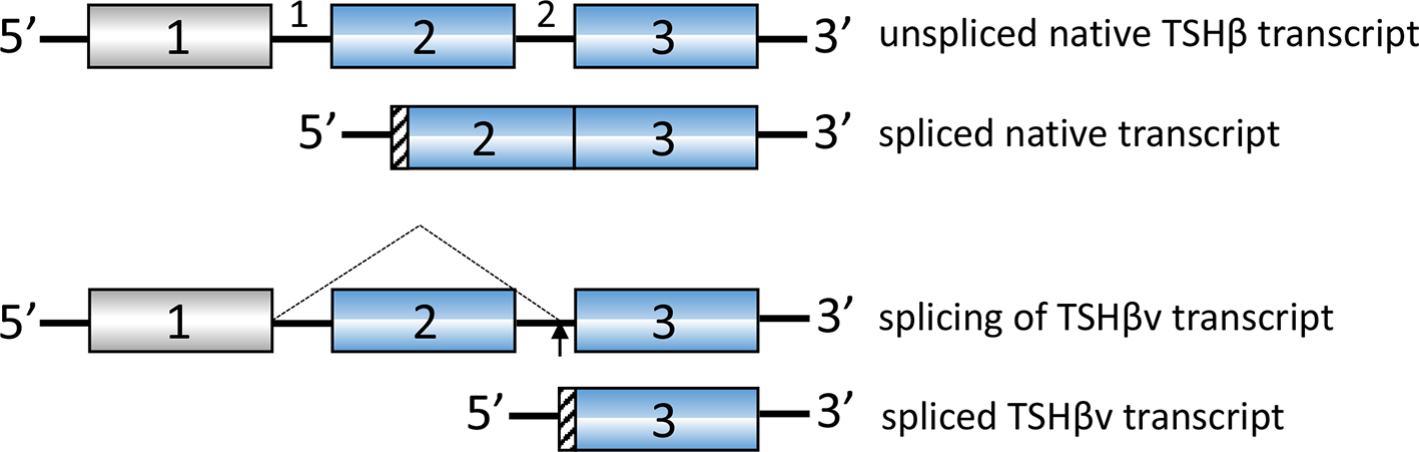
Putative splicing mechanism used to generate human TSHβv in cells of the immune system. Donor splice sites in intron 1 and acceptor splice sites in intron 2 remove exon 2. A portion of intron 2 is used for the signal peptide as shown in **Figure 1**.

TSHβ is coded for by exons 2 and 3 in humans and exons 4 and 5 in mice. The splice variant is unique, however, in that in both species only the second of the two exons is used to code for TSHβv, with a small portion of the upstream intron coding for a signal peptide (**Figure 1**). Predictions as to the mechanisms of alternative splicing of TSHβ in leukocytes leading to the generation of TSHβv are derived from putative donor and acceptor splice sites in human intron 1 and intron 2, respectively, resulting in the elimination of exon 2 and the retention of an intron 2 associated signal peptide (**Figure 2**) (41).

TSHβv has been shown to be present in the human circulation (42), and to be functionally active based on cAMP signaling (8, 39) as well as to successfully couple to TSHα (42), a condition considered to be essential to achieve full biological activity (43). Moreover, TSHβv has been shown to induce TH synthesis *in vivo* and *in vitro*. T3 and T4 were elevated in the circulation of mice within one hour of injection of recombinant TSHβv, and to induce the secretion of T3 and T4 from thyroid follicular cells *in vitro* (44). What’s more, levels of thyroglobulin, thyroid peroxidase, and sodium-iodide supporter were elevated in thyroid follicular cells following TSHβv stimulation. Of particular interest, injection of mice with T3 and TRH caused a transient drop followed by an increase in native TSHβ though not in TSHβv in the pituitary (44).

**Figure 3:**
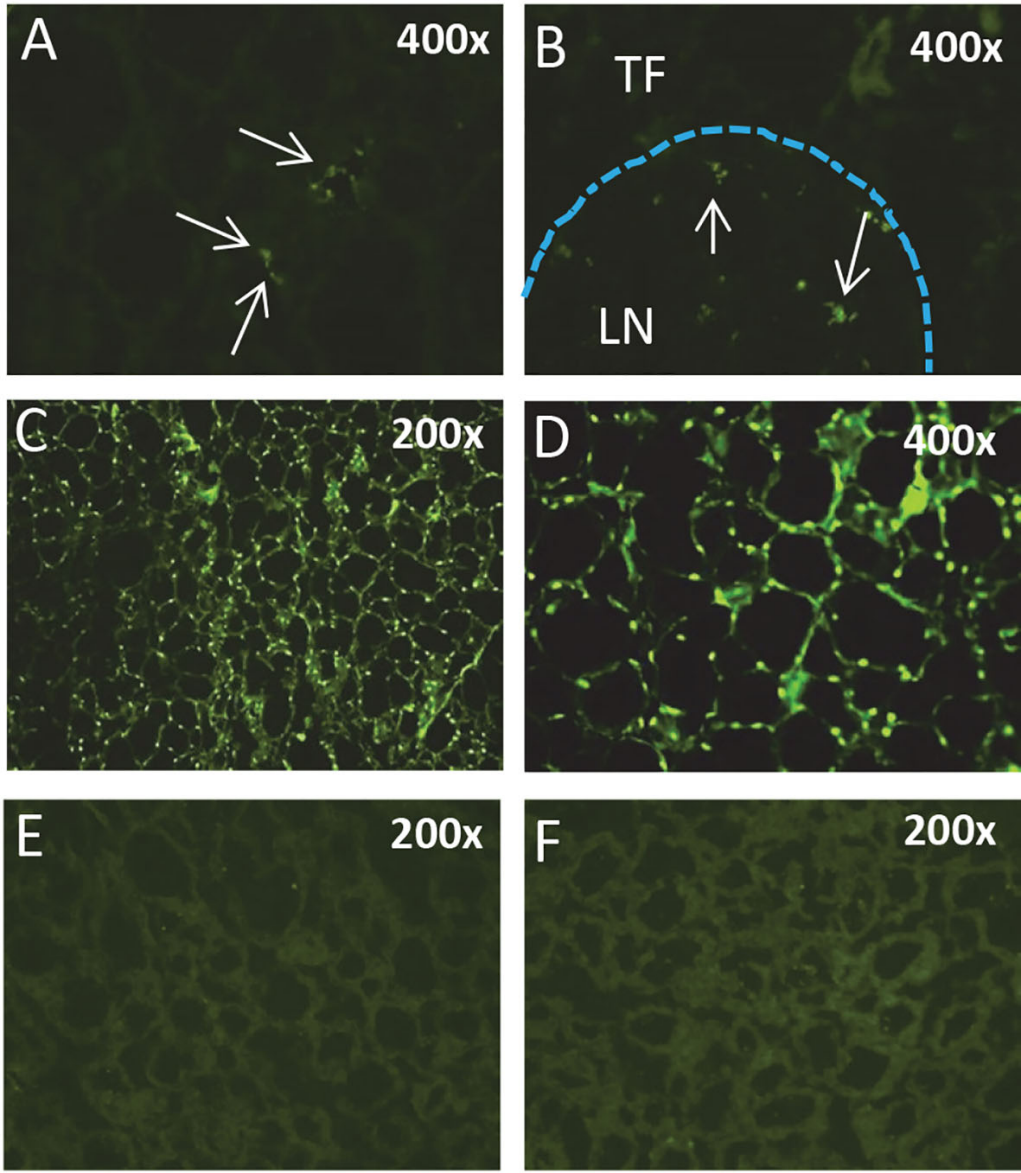
Splenic leukocytes from *L. monocytogenes*-infected mice but not normal mice traffic to the thyroid. Immunofluorescence analysis of **(A)** the thyroid and **(B)** a thyroid perivascular lymph node from a non-infected mouse 24 hours post-cell transfer of CFSE-labeled splenic leukocytes from a *L. monocytogenes*-infected mouse. **(C, D)** Thyroid of a non-infected mouse 48 hours post-transfer of spleen cells from a *L. monocytogenes*-infected mouse. CFSE-labeled leukocytes are present surrounding thyroid follicles. **(E, F)** Thyroid of a non-infected mouse injected with CFSE-labeled spleen cells from a non-infected mouse. TF, thyroid follicle; LN, lymph node.

Expression of TSHβv has been linked to the inflammatory response in AIT, in particular in Hashimoto’s thyroiditis (HT), as demonstrated by elevated transcript levels of TSHβv in PBL of patients with HT compared to normal controls (42). Treatment of patients with prednisone reduced TSHβv transcript levels in persons with short duration of disease compared to persons with long duration. Additionally, TSHβv-producing plasma cells infiltrated the thyroid in HT patients (40). Recent studies demonstrate that immune system TSHβv in humans operates independently of the HPT axis and is capable of inducing TH synthesis from PBL in times of immune stress, such as during systemic infection (44). Those possibility conforms to finding in mice showing that TSHβv-producing inflammatory cells traffic to the thyroid following *L. monocytogenes* infection (38). Moreover, spleen cells from bacteria-infected mice, but not from non-infected mice, trafficked to the thyroid of normal non-infected mice at high density 48 hours post-transfer (**Figure 3**) (38). The connection between infection and AIT, while interesting, is unclear due in part to a lack of sufficient studies to draw definitive conclusions (45). Taken together, however, these findings suggest that under certain conditions TSHβv may contribute to the pathogenesis of HT and possibly other forms of AIT.

TSH has been shown to directly influence bone remodeling *via* TSHR expressed on osteoclasts by preventing bone resorption (46) and stimulating osteoblastic bone formation (47). In humans, there is an increased risk of bone fracture in women with low circulating TSH (48). Using *Tshr^-/-^* mice, which are incapable of TSH signaling, and WT mice that were induced to a state of hyperthyroidism by implantation of T4 pellets, *Tshr^-/-^* mice had significantly greater bone loss (49), further suggesting a role for TSH in bone restructuring. Moreover, expression of TSHβv in BM CD11b^+^ cells was positively rather than negatively regulated by *in vivo* T3 supplementation (49). This was further confirmed using human BM-derived macrophages, which had an increase in TSHβv following exposure to T3 in a dose-dependent manner (50). Those findings further indicate that the regulation of TSHβv by TH occurs independently of HPT axis control.

## Summary and Conclusions

Over the past forty years, a large body or information has come forth defining an intricate nexus between the immune system and the endocrine system. Immune-endocrine pathways have effects on normal as well as pathophysiological processes, some of which is mediated by a novel alternatively-spliced form of TSHβ produced by the hematopoietic system. Indeed, a number of studies remain to be done to fully understand the biological implications of immune system TSHβ cell signaling in the thyroid and bone. For example, the extent to which native TSH and TSHβv work synergistically or antagonistically in delivering TSHR-mediated signals may provide important information into the specific role of TSHβ in AIT and osteoporosis.

## Author Contributions

JK is the sole author to all aspects of this article.

## Funding

This work was funded in part by NIH grants R21AI135293 and R21 AI133313.

## Conflict of Interest

The author declares that the research was conducted in the absence of any commercial or financial relationships that could be construed as a potential conflict of interest.

## References

[1] Blalock JE. The immune system as the sixth sense. J Intern Med (2005) 257:126–38. 10.1111/j.1365-2796.2004.01441.x 15656872

[2] Furness JB Kunze WA Clerc N. Nutrient tasting and signaling mechanisms in the gut. II. The intestine as a sensory organ: neural, endocrine, and immune responses. Am J Physiol (1999) 277:G922–928. 10.1152/ajpgi.1999.277.5.G922 10564096

[3] Smith EM Phan M Kruger TE Coppenhaver DH Blalock JE. Human lymphocyte production of immunoreactive thyrotropin. Proc Natl Acad Sci USA (1983) 80:6010–3. 10.1073/pnas.80.19.6010 PMC534349 6351072

[4] Kruger TE Smith LR Harbour DV Blalock JE. Thyrotropin: an endogenous regulator of the in vitro immune response. J Immunol (1989) 142:744–7. 2492328

[5] Scofield VL Montufar-Solis D Cheng E Estes MK Klein JR. Intestinal TSH production is localized in crypt enterocytes and in villus ‘hotblocks’ and is coupled to IL-7 production: evidence for involvement of TSH during acute enteric virus infection. Immunol Lett (2005) 99:36–44. 10.1016/j.imlet.2004.12.010 15894109 PMC2894696

[6] Klein JR. The immune system as a regulator of thyroid hormone activity. Exp Biol Med (2006) 231:229–36. 10.1177/153537020623100301 PMC2768616 16514168

[7] Bagriacik EU Zhou Q Wang HC Klein JR. Rapid and transient reduction in circulating thyroid hormones following systemic antigen priming: implications for functional collaboration between dendritic cells and thyroid. Cell Immunol (2001) 212:92–100. 10.1006/cimm.2001.1846 11748925

[8] Vincent BH Montufar-Solis D Teng BB Amendt BA Schaefer J Klein JR. Bone marrow cells produce a novel TSHb splice variant that is upregulated in the thyroid following systemic virus infection. Genes Immun (2009) 10:18–26. 10.1038/gene.2008.69 18754015 PMC2629504

[9] Visser WE Friesema EC Jansen J Visser TJ. Thyroid hormone transport in and out of cells. Trends Endocrinol Metab (2008) 19:50–6. 10.1016/j.tem.2007.11.003 18291666

[10] Bianco AC Salvatore D Gereben B Berry MJ Larsen PR. Biochemistry, cellular and molecular biology, and physiological roles of the iodothyronine selenodeiodinases. Endocr Rev (2002) 23:38–89. 10.1210/edrv.23.1.0455 11844744

[11] Williams GR. Extrathyroidal expression of TSH receptor. Ann Endocrinol (Paris) (2011) 72:68–73. 10.1016/j.ando.2011.03.006 21511243

[12] Savino W Wolf B Aratan-Spire S Dardenne M. Thymic hormone containing cells. IV. Fluctuations in the thyroid hormone levels in vivo can modulate the secretion of thymulin by the epithelial cells of young mouse thymus. Clin Exp Immunol (1984) 55:629–35. PMC1535945 6584256

[13] Fabris N Mocchegiani E. Endocrine control of thymic serum factor production in young-adult and old mice. Cell Immunol (1985) 91:325–35. 10.1016/0008-8749(85)90230-8 4039631

[14] Fabris N Mocchegiani E Mariotti S Pacini F Pinchera A. Thyroid function modulates thymic endocrine activity. J Clin Endocrinol Metab (1986) 62:474–8. 10.1210/jcem-62-3-474 3944232

[15] Fabris N Mocchegiani E Mariotti S Pacini F Pinchera A. Thyroid-thymus interactions during development and aging. Horm Res (1989) 31:85–9. 10.1159/000181093 2722140

[16] Barreiro Arcos ML Sterle HA Paulazo MA Valli E Klecha AJ Isse B. Cooperative nongenomic and genomic actions on thyroid hormone mediated-modulation of T cell proliferation involve up-regulation of thyroid hormone receptor and inducible nitric oxide synthase expression. J Cell Physiol (2011) 226:3208–18. 10.1002/jcp.22681 21344381

[17] Barreiro Arcos ML Sterle HA Vercelli C Valli E Cayrol MF Klecha AJ. Induction of apoptosis in T lymphoma cells by long-term treatment with thyroxine involves PKCzeta nitration by nitric oxide synthase. Apoptosis (2013) 18:1376–90. 10.1007/s10495-013-0869-8 23733107

[18] Sterle HA Valli E Cayrol F Paulazo MA Martinel Lamas DJ Diaz Flaque MC. Thyroid status modulates T lymphoma growth via cell cycle regulatory proteins and angiogenesis. J Endocrinol (2014) 222:243–55. 10.1530/JOE-14-0159 24928937

[19] Mihara S Suzuki N Wakisaka S Suzuki S Sekita N Yamamoto S. Effects of thyroid hormones on apoptotic cell death of human lymphocytes. J Clin Endocrinol Metab (1999) 84:1378–85. 10.1210/jcem.84.4.5598 10199782

[20] Montecino-Rodriguez E Clark R Johnson A Collins L Dorshkind K. Defective B cell development in Snell dwarf (dw/dw) mice can be corrected by thyroxine treatment. J Immunol (1996) 157:3334–40. 8871629

[21] Perrotta C Buldorini M Assi E Cazzato D De Palma C Clementi E. The thyroid hormone triiodothyronine controls macrophage maturation and functions: protective role during inflammation. Am J Pathol (2014) 184:230–47. 10.1016/j.ajpath.2013.10.006 24215914

[22] Chen Y Sjolinder M Wang X Altenbacher G Hagner M Berglund P. Thyroid hormone enhances nitric oxide-mediated bacterial clearance and promotes survival after meningococcal infection. PloS One (2012) 7:e41445. 10.1371/journal.pone.0041445 22844479 PMC3402396

[23] Al-Abed Y Metz CN Cheng KF Aljabari B VanPatten S Blau S. Thyroxine is a potential endogenous antagonist of macrophage migration inhibitory factor (MIF) activity. Proc Natl Acad Sci U S A (2011) 108:8224–7. 10.1073/pnas.1017624108 PMC3100930 21536912

[24] Calandra T Echtenacher B Roy DL Pugin J Metz CN Hultner L. Protection from septic shock by neutralization of macrophage migration inhibitory factor. Nat Med (2000) 6:164–70. 10.1038/72262 10655104

[25] Bagriacik EU Klein JR. The thyrotropin (thyroid-stimulating hormone) receptor is expressed on murine dendritic cells and on a subset of CD45RBhigh lymph node T cells: functional role for thyroid-stimulating hormone during immune activation. J Immunol (2000) 164:6158–65. 10.4049/jimmunol.164.12.6158 10843665

[26] Dedecjus M Stasiolek M Brzezinski J Selmaj K Lewinski A. Thyroid hormones influence human dendritic cells’ phenotype, function, and subsets distribution. Thyroid (2011) 21:533–40. 10.1089/thy.2010.0183 21190445

[27] Ouchi N Parker JL Lugus JJ Walsh K. Adipokines in inflammation and metabolic disease. Nat Rev Immunol (2011) 11:85–97. 10.1038/nri2921 21252989 PMC3518031

[28] Tilg H Moschen AR. Adipocytokines: mediators linking adipose tissue, inflammation and immunity. Nat Rev Immunol (2006) 6:772–83. 10.1038/nri1937 16998510

[29] Wolf AM Wolf D Avila MA Moschen AR Berasain C Enrich B. Up-regulation of the anti-inflammatory adipokine adiponectin in acute liver failure in mice. J Hepatol (2006) 44:537–43. 10.1016/j.jhep.2005.08.019 16310276

[30] Maeda N Shimomura I Kishida K Nishizawa H Matsuda M Nagaretani H. Diet-induced insulin resistance in mice lacking adiponectin/ACRP30. Nat Med (2002) 8:731–7. 10.1038/nm724 12068289

[31] Wensveen FM Sestan M Turk Wensveen T Polic B. ‘Beauty and the beast’ in infection: How immune-endocrine interactions regulate systemic metabolism in the context of infection. Eur J Immunol (2019) 49:982–95. 10.1002/eji.201847895 31106860

[32] Loffreda S Yang SQ Lin HZ Karp CL Brengman ML Wang DJ. Leptin regulates proinflammatory immune responses. FASEB J (1998) 12:57–65. 10.1096/fasebj.12.1.57 9438411

[33] Glass CK Olefsky JM. Inflammation and lipid signaling in the etiology of insulin resistance. Cell Metab (2012) 15:635–45. 10.1016/j.cmet.2012.04.001 PMC4156155 22560216

[34] Lumeng CN Saltiel AR. Inflammatory links between obesity and metabolic disease. J Clin Invest (2011) 121:2111–7. 10.1172/JCI57132 PMC3104776 21633179

[35] Lynch L Hogan AE Duquette D Lester C Banks A LeClair K. iNKT Cells Induce FGF21 for Thermogenesis and Are Required for Maximal Weight Loss in GLP1 Therapy. Cell Metab (2016) 24:510–9. 10.1016/j.cmet.2016.08.003 PMC5061124 27593966

[36] Finlin BS Zhu B Confides AL Westgate PM Harfmann BD Dupont-Versteegden EE. Mast Cells Promote Seasonal White Adipose Beiging in Humans. Diabetes (2017) 66:1237–46. 10.2337/db16-1057 PMC5399616 28250021

[37] Coquet JM Chakravarti S Kyparissoudis K McNab FW Pitt LA McKenzie BS. Diverse cytokine production by NKT cell subsets and identification of an IL-17-producing CD4-NK1.1- NKT cell population. Proc Natl Acad Sci U S A (2008) 105:11287–92. 10.1073/pnas.0801631105 PMC2516267 18685112

[38] Montufar-Solis D Klein JR. Splenic Leukocytes Traffic to the Thyroid and Produce a Novel TSHbeta Isoform during Acute Listeria monocytogenes Infection in Mice. PloS One (2016) 11:e0146111. 10.1371/journal.pone.0146111 26771831 PMC4714905

[39] Baliram R Chow A Huber AK Collier L Ali MR Morshed SA. Thyroid and Bone: Macrophage-Derived TSH-beta Splice Variant Increases Murine Osteoblastogenesis. Endocrinology (2013) 154:4919–26. 10.1210/en.2012-2234 PMC3836071 24140716

[40] Liu CR Miao J Zhao ZK Li LY Liu YM Zhang YL. Functional human TSHbeta splice variant produced by plasma cell may be involved in the immunologic injury of thyroid in the patient with Hashimoto’s thyroiditis. Mol Cell Endocrinol (2015) 414:132–42. 10.1016/j.mce.2015.06.009 26170068

[41] Klein JR. Novel Splicing of Immune System Thyroid Stimulating Hormone beta-Subunit-Genetic Regulation and Biological Importance. Front Endocrinol (Lausanne) (2019) 10:44. 10.3389/fendo.2019.00044 30804891 PMC6371030

[42] Liu C Li L Ying F Xu C Zang X Gao Z. A newly identified TSHbeta splice variant is involved in the pathology of Hashimoto’s thyroiditis. Mol Biol Rep (2012) 39:10019–30. 10.1007/s11033-012-1871-x 22752807

[43] Szkudlinski MW Fremont V Ronin C Weintraub BD. Thyroid-stimulating hormone and thyroid-stimulating hormone receptor structure-function relationships. Physiol Rev (2002) 82:473–502. 10.1152/physrev.00031.2001 11917095

[44] Liu C Miao J Liu X Zhao Z Kou T Liu J. HPT axisindependent TSHbeta splice variant regulates the synthesis of thyroid hormone in mice. Mol Med Rep (2019) 19:4514–22. 10.3892/mmr.2019.10082 30942410

[45] Davies TF. Infection and autoimmune thyroid disease. J Clin Endocrinol Metab (2008) 93:674–6. 10.1210/jc.2008-0095 18326006

[46] Abe E Marians RC Yu W Wu XB Ando T Li Y. TSH is a negative regulator of skeletal remodeling. Cell (2003) 115:151–62. 10.1016/S0092-8674(03)00771-2 14567913

[47] Sampath TK Simic P Sendak R Draca N Bowe AE O’Brien S. Thyroid-stimulating hormone restores bone volume, microarchitecture, and strength in aged ovariectomized rats. J Bone Miner Res (2007) 22:849–59. 10.1359/jbmr.070302 17352644

[48] Bauer DC Ettinger B Nevitt MC Stone KL Study of Osteoporotic Fractures Research, G. Risk for fracture in women with low serum levels of thyroid-stimulating hormone. Ann Internal Med (2001) 134:561–8. 10.7326/0003-4819-134-7-200104030-00009 12803168

[49] Baliram R Sun L Cao J Li J Latif R Huber AK. Hyperthyroid-associated osteoporosis is exacerbated by the loss of TSH signaling. J Clin Invest (2012) 122:3737–41. 10.1172/JCI63948 PMC3461920 22996689

[50] Baliram R Latif R Morshed SA Zaidi M Davies TF. T3 Regulates a Human Macrophage-Derived TSH-beta Splice Variant: Implications for Human Bone Biology. Endocrinology (2016) 157:3658–67. 10.1210/en.2015-1974 PMC5007892 27300765

